# The Efficacy of Parent Training Interventions with Parents of Children with Developmental Disabilities

**DOI:** 10.3390/ijerph19159685

**Published:** 2022-08-05

**Authors:** Benedetta Ragni, Francesca Boldrini, Sonia Mangialavori, Marco Cacioppo, Michele Capurso, Simona De Stasio

**Affiliations:** 1 Department of Human Studies, LUMSA University, Piazza delle Vaschette, 101, 00193 Rome, Italy; 2 Department of Pathophysiology and Transplantation, University of Milano, 20122 Milan, Italy; 3 Department of Philosophy, Social Sciences & Education, University of Perugia, 06123 Perugia, Italy

**Keywords:** parenting, developmental disabilities, infancy, parent training, intervention

## Abstract

Parenting children with developmental disabilities (DD) can be generally characterized by a considerable psychological burden. The effects on parental and familial psychological well-being and, consequently, on children’s developmental outcomes should not be underestimated, especially in early childhood. The current review aims to advance our understanding of the key factors (e.g., formats, sample characteristics, research design) that characterize parent training interventions, and that could be related to their outcomes, to guide researchers and clinical practitioners to develop and provide efficient programs. Studies were identified via an Internet search from three electronic databases, following PRIMSA guidelines. Studies published until November 2021 were taken into account. The initial search yielded a total of 2475 studies. Among them, 101 studies were fully reviewed. Finally, ten of the studies, which met all the inclusion criteria, formed the basis for this review. Participants’ characteristics, main features of the interventions (i.e., study design, structure, and contents), outcome variables and treatment efficacy were deeply examined and discussed. Key factors of parent training interventions with parents of children affected by DD are enlightened, to guide researchers and clinicians in the design and implementation of tailored specific programs, aimed to sustain parenting and foster children’s developmental outcomes, from early stages of life.

## 1. Introduction

Over the past decades, global rates of chronic diseases and disabilities throughout infancy and childhood have increased progressively, despite improved survival rates for rare and complex conditions. Better awareness, rather than an effective increase in prevalence, and the development of more sensitive tools enabling early diagnosis, have led to increased diagnoses of developmental disabilities (DD) [1,2]. The Diagnostic and Statistical Manual of Mental Disorders [3] defines “Developmental Disabilities” (DD) as intellectual disabilities, communication disorders, autism spectrum disorders, and motor disorders. Besides, according to the International Classification of Diseases [4], DD comprise several conditions: mental retardation, specific speech and language disorders, pervasive developmental disorders, and specific developmental disorders of the motor functions. These life-long conditions are characterized by early-onset during infancy and an overall delay in the development of the central nervous system. Considerable functional limitations in multiple domains, including learning, mobility, self-care, and autonomy, are reported. Compared to typically developed peers, children with DD show an increased risk for emotional, internalizing, and externalizing behavior comorbidities [5]. In particular, behavioral problems can emerge during infancy, persist in adolescence, and endure throughout the later stages of life [6]. The psychological burden associated with the diagnosis and its impact on parental and familial wellbeing is considerable and should not be underestimated [7]. Nurturing a child with special care needs can be extremely challenging. As [8] described, intensive support from caregivers is inevitably required to guarantee their physical, social, and emotional basic needs and foster a positive quality of life. Also crucial is substantial mutual cooperation between parents [9,10]. Research comparing parenting children with developmental disabilities and typically developed children outlined the differences between the two groups. First, significant stress levels characterize parenting children with a diagnosis, particularly in mothers [11,12,13,14,15]. In addition, global parental satisfaction with caregiving is affected following a diagnosis of DD; parents of the clinical group experienced lower levels of satisfaction than their counterparts with typically developing children [16]. The literature widely reports how positive parenting and positive parent-child interactions support and foster children’s wellbeing, sustaining adaptive adjustments and psychosocial development. In families with children affected by DD, positive parenting behavior is consistently associated with positive child outcomes, quality of parental support in children’s activities, increased capacity for independent living, development of social skills, and reductions in challenging behaviors [17,18,19].

On this premise, effective prevention and treatment interventions supporting caregivers are essential to reduce parental strain and effort [20,21]. Early childhood parent training programs are effective and show promise in supporting families facing different risks [22]. The possibility for parents of children with DD to access therapeutic interventions in the early stages of life can considerably improve the children’s long-term outcomes, encouraging favorable developmental pathways [23]. The current literature cites several parent training programs for parents of children with DD [24]. However, while similar in content, these studies differ in their delivery, the methodology, and the research design. Meta-analyses on the efficacy of parent management training to reduce child behavioral difficulties and parental stress in families of typically developed children [25] have highlighted that heterogeneity in the intervention formats (e.g., self-administered, group sessions), in the children’s characteristics (e.g., age), and parent and community factors (e.g., mental health, socioeconomic status (SES), and social support) could influence the treatments’ outcomes. Consequently, it is essential to understand and clarify whether these factors could also be related to the treatment response in families of children with DD to provide interventions that are as individualized as possible and based on each family’s particular needs and characteristics.

For the reasons stated above, we systematically assessed the existing literature on parent training interventions targeting parents of children with DD aged from 0–6 years. Specifically, the current review aims to advance our understanding of the key factors (e.g., formats, sample characteristics, research design) that characterize these parent training interventions and that could be related to their outcomes to guide researchers and clinical practitioners to develop and provide effective and tailored programs that could foster the specific needs of parents and children.

## 2. Materials and Methods

In planning and conducting this study, we followed the guidelines from the Preferred Reporting Items for Systematic Reviews and Meta-Analyses (PRISMA) statement [26]. Heterogeneity in the studies and in the authors’ reporting of outcomes, and a lack of detailed statistical information in many studies, precluded a meta-analysis. Therefore, the authors adopted a systematic narrative approach to report the study’s key findings. First, we identified existing studies on parent training interventions aimed at helping parents of children with DD to prevent and manage their children’s challenging behaviors. Specifically, we investigated parent training intervention studies whose outcomes led to changes in both parental and children’s behaviors. According to the literature, supporting parenting skills reduces the risk of later problem behaviors in children with DD and supports family wellbeing and parental mental health [27]. For this reason, we adopted the following inclusion criteria: (a) empirical studies published in peer-reviewed, English-language scientific journals; and (b) studies with samples comprising parents of children diagnosed with DD. In this context, DD refer to conditions classified in the Diagnostic and Statistical Manual of Mental Disorders [3] as intellectual disabilities, communication disorders, autism spectrum disorders, and motor disorders, and in the International Classification of Diseases [4] as mental retardation, specific disorders of speech and language, pervasive developmental disorder, and specific developmental disorders of motor functions. The parents of these children experience similar stressors. The aims and nature of the interventions analyzed were to improve parental and child wellbeing, rather than providing specific instructions on how to intervene with behaviors related to specific diagnoses such as autistic spectrum disorder (ASD), attention deficit hyperactivity disorder (ADHD), etc. [28]; (c) studies with samples comprising parents of children aged 0–6 years. Moreover, these interventions should have been (d) implemented and (e) include changes in parental and children’s adjustment/behaviors as outcome variables. 

The studies, published until November 2021, were identified via an Internet search of the SCOPUS, PubMed, and PsycINFO electronic databases. We adopted an iterative search strategy with three sets of terms: (“developmental disabilit* OR “developmental delay*”) AND (“parent*”) AND (“intervention*” OR “training*” OR “program*” OR “tool*”). We excluded: intervention studies (a) with samples comprising parents of children aged > 6 years; (b) samples including caregivers other than parents (e.g., grandparents); (c) interventions that were not implemented and empirically evaluated studies (e.g., protocol studies); (d) studies that did not measure both parental and children’s outcome variables; (e) studies created and validated only for parents of children with specific diagnoses; (f) studies that included parent training as a component of a multifaceted treatment were excluded if the effects of the parent training could not be isolated (McIntyre, 2013); (g) grey literature; and (h) articles whose full text could not be accessed. 

**Figure 1:**
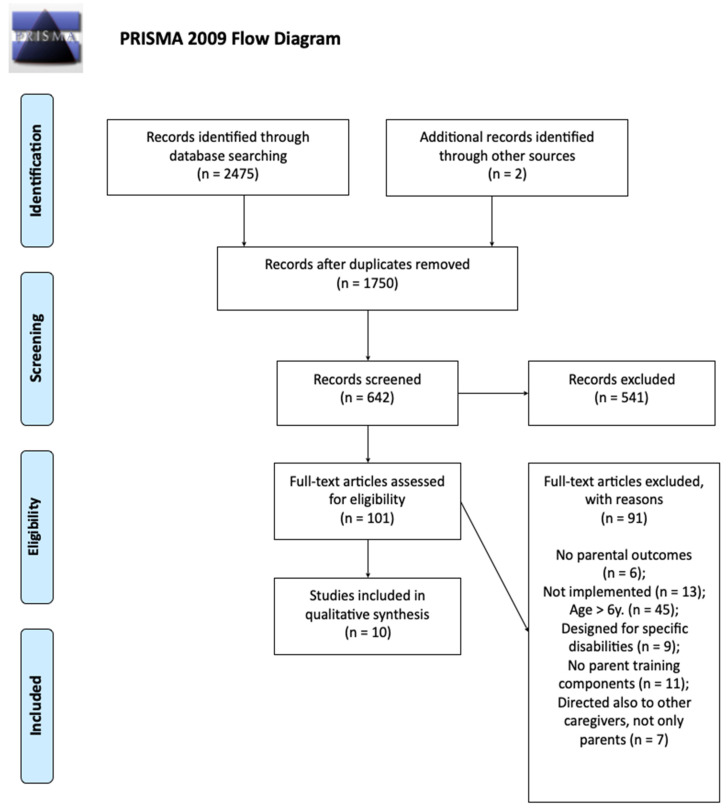
Flowchart for the systematic review procedure.

The flowchart for the systematic review procedure is displayed in Figure 1.

The initial search yielded 2475 studies. After eliminating duplicates, 1750 remained. Following an initial check of the titles and abstracts, 541 studies were rejected, as they did not meet the inclusion criteria, leaving 101 studies to be read thoroughly. Finally, ten studies met all the inclusion criteria and formed the basis for the review. Detailed information was drawn from each relevant article using a researcher-developed data extraction sheet. The following areas were included: (1) authors, year of publication, and country of data collection; (2) information on the implemented program (name of the program, whether it was validated, or if it was adapted from other validated programs); (3) sample characteristics: number of parents enrolled, number of mothers/fathers enrolled, children’s age, and SES background; (4) intervention characteristics: main content of each intervention, research design (randomized controlled trial, pre-post design, follow-up evaluations), in-person/web-based intervention, if the intervention included a group and/or individual training, activities and duration of training, if the intervention included homework for parents, follow-up activities after the intervention, and a control group; (5) measured variables: outcome variables (evaluated with structured observation or with self-report questionnaires), covariates, moderators, acceptability/satisfaction with the program reported by participants; and (6) main results. Four authors coded the data, and the coding procedure was refined via consensus discussions. More specifically, the first five articles were randomly chosen for coding. Discrepancies were resolved via joint reviews and discussions, and minor adjustments were made to the data extraction sheet. The authors then extracted data from ten articles each, and accuracy was jointly assessed by all three of the author-judges. The information extracted from the set of relevant articles is summarized in Table A1 and Table A2 (See Appendix A).

The study was conducted according to the guidelines of the Declaration of Helsinki and approved by the Institutional Review Board of LUMSA University (protocol code CERS07052020 approved on 14 May 2020).

## 3. Results

The ten studies included in the final review were conducted in three countries: The USA (6), China (2), and Australia (2). All the articles were published between 1993 and 2018. Five studies implemented a validated program, three examined an adapted version of a validated program and two contained novel interventions. Table A1 shows the characteristics of each intervention reviewed. 

### 3.1. Participants Characteristics 

The number of enrolled parents ranged from a minimum of two to a maximum of 201, and the mean number was 56. Eight of the ten studies reported the gender of caregivers, with all focusing primarily or exclusively on the mothers. In addition, four studies included families from different SES backgrounds, while one study examined upper-middle-class families only (five studies did not report information on children’s SES). The children’s ages ranged from 34 to 57 months, with a mean age of 49 months. The children’s disability status varied across the ten studies. In five studies, children were evaluated by experts and enrolled if they had a mild to moderate disability [29,30,31,32], or a moderate to severe disability [33]. The remaining five studies included multiple DD conditions [34,35,36,37,38]. The presence of children’s challenging behaviors was an inclusionary criterion in three studies (30%) [29,34,37] and one study [32] included only mothers who displayed negative parenting strategies and low levels of positive parenting strategies (i.e., less than a 3:1 ratio of positive to negative strategies) at the baseline assessment of the observed parent–child interactions for more than 20% of the observed intervals.

### 3.2. Intervention Characteristics 

#### 3.2.1. Study Design

Six studies used a randomized controlled trial with a control group to evaluate the effects of parent training interventions on the outcomes for parents and children. Specifically, four studies used a waitlist control group, and two used a usual care control group. Among these studies, Phaneuf and McIntyre [32] used a changing conditions design in which they increased the intensity of the parent training along three tiers, depending on the parental response to the intervention. However, Plant and Sanders [37] compared two experimental groups (standard and enhanced Stepping Stones Triple P (SSTP) with a waitlist control group. Finally, while all the studies performed evaluation pre-and post-treatment, only four included a follow-up evaluation from six weeks to one year after the post-test measures.

#### 3.2.2. Structure and Contents

As shown in Table A1, five (50%) parent training studies used group formats to deliver the intervention [30,31,33,35,38], while one study used individual one-to-one sessions [29] and four studies reported delivering parent training using multiple formats [32,34,36,37]. Interventions were all therapist-delivered programs. Only one study also included a self-administered session [32]. All the studies reported using treatment manuals or protocols to guide the parent training interventions. The parent training interventions included in this review were similar. Specifically, six validated interventions were used as described below. Five studies used the Incredible Years Parent Training program (IYPT) [24] with parents of children with DD. Specifically, Barton and Lissman [34] used a shorter version, and McIntyre [30,31,32] used an adapted version of the Incredible Years with parents of children with DD (IYPT-DD) [30]. Kong and Au culturally adapted the IYPT and IYPT-DD contents for Chinese parents [35]. IYPT is an evidence-based parent training program based on the principles of operant and social learning theories [39]. It is delivered in 12 weekly sessions. 

Group leaders use discussion, video modeling, role-playing, and didactics to cover topics in five main areas: play, praise, rewards, limit setting, and handling challenging behavior. Challenging behavior is reduced by altering negative and coercive parent–child interactions [24]. DD modifications implemented by McIntyre (IYPT-DD) included discussing the unique challenges associated with raising a child with DD, understanding children’s developmental levels and support needs, conducting descriptive functional behavioral assessments, and developing behavior support plans based on the hypothesized function of the child’s challenging behavior [30]. Of the five studies that implemented the IYPT and IYPT-DD, only [32] changed the duration and structure of the intervention. They implemented a three-tiered model of interventions that increased the intensity of support depending on the parents’ responsiveness to the intervention. The three intervention tiers evaluated by Phaneuf and McIntyre included self-administered reading materials, group-based parenting training based on the Incredible Years with DD modifications (IYPT-DD), and individualized video feedback based on the behavioral skills training literature [40] with content covering the IYPT-DD [41].

One study implemented the Triple P program [36], and another [37] reported on the effects of SSTP in families of children with DD. Stepping Stones is an alternative version of the Triple P Positive Parenting Program [42] and was created for use with parents of children with disabilities. Like the IYPT, the Triple P is based on operant and social learning theories and draws on principles guided by applied behavior analysis to reduce problematic child behaviors by interventions of the parent–child relationship [42]. SSTP includes components of the original Triple P program (e.g., reinforcement-based approaches to increase positive behavior, differential reinforcement to decrease challenging behavior, and consideration of the function of the problem behavior) plus strategies from special education literature (e.g., skill acquisition and functional communication training). In addition to this, Plant and Sanders [37] compared the traditional SSTP with an enhanced version, the SSTP-E. SSTP-E consisted of the SSTP with additional training on grief and loss, stress and coping, time management, working collaboratively with professionals, and strengthening social support.

One study [38] reported on the use of Sing & Grow, a 10-week group-based early childhood parenting intervention that uses music-based play activities to enhance responsive parenting and promote child development [43]. Sing & Grow is delivered by trained music therapists and is based on attachment theory and behavioral parent training interventions, such as the Triple P Positive Parenting Program [42]. Sing & Grow uses music to enhance parent–child relationships. Improvements in child development are thought to be promoted by strengthening the quality of the parent–child relationship [43]. Parent behaviors targeted in this intervention include parental expression of affection, physical touch, praise, appropriate instruction-giving, parental emotional responsiveness, and confidence-building in parenting skills [38,44]. Through their participation in Sing & Grow, parents learn about the developmental needs of their children and have more appropriate expectations for their children’s development and the management of challenging behaviors.

One study by Bagner and Eyberg [29] reported on the use of the Parent–Child Interaction Therapy (PCIT) for children with DD and oppositional defiant disorder. PCIT is an evidence-based parent training intervention based on attachment and social learning theories [45] aimed at reducing children’s disruptive behaviors by fostering positive parent–child interactions. PCIT is individually delivered and comprises two phases: a child-directed intervention (CDI) phase and a parent-directed intervention (PDI) phase. The CDI training focuses on increasing positive interactions between the parent and child through play and praise. The PDI focuses on increasing children’s compliance and decreasing their aggressive behaviors through limit-setting strategies [45].

Finally, the last intervention implemented included in this review [33], the Parents Involved in Education (PIE) curriculum, is a parent training program designed to teach parents strategies they can implement at home to prepare them to interact more effectively with professional intervenors, teach them basic child development principles, provide opportunities for parents to discuss challenges associated with parenting a child with a disability, and facilitate support networks.

#### 3.2.3. Outcome Variables and Measures

Table A2 includes outcome variables, measures, and results for each of the interventions analyzed.

All but one study [33] included parent-reported measures of children’s behaviors, and nine studies included direct observations of child behaviors led by experts. Three studies included parent-reported measures of parental behaviors and parenting styles [36,37,38], and nine studies measured parental behaviors through direct observations conducted by experts [29,30,31,32,33,34,35,37,38]. One study [36] included only self-reported measures, and Boyce et al. involved teachers in evaluating the children’s development and the parental grade of participation in the program [33]. 

Other measures rated by parents and included as dependent variables were: the impact on the child’s family [30,31], parental competence/self-efficacy [34,37,38], parental stress [29,35,36,37], parental depression [31,37], parental anxiety [37], general parental mental health [38], child development (also rated by experts and teachers [33]), family functioning [33], conflicts between partners over child-rearing [36], quality of dyadic relationship adjustments [37], and perceived social support from the other parents in the group [38].

Alongside the dependent variables, three studies tested the role of covariates [30,33,38] and one study [29] tested a mediator variable to predict pre-post behavioral changes in the children. Specifically, the covariates were: socio-demographic factors (mothers’ years of education, fathers’ hours of work, child age and gender, the main language used at home, main income, being a single parent), high parental attendance at the intervention sessions, children’s general health, changes in family living situations and stressful life events, family resources, the presence of a support person during the intervention and type of DD (autism vs. other DD). Meanwhile, Bagner and Eyberg tested the mediation role of the pre-post changes in maternal parenting skills on pre-post changes in child behaviors [29].

#### 3.2.4. Treatment Efficacy

Nine studies showed a reduction in the children’s challenging behaviors. Specifically, four studies identified changes in the children’s behaviors measured by parent-reposted measures (also at the six-month follow-up evaluation [29,34,35,36]) and two studies were measured by parental self-report measures and direct observation by experts [32,37]. Three studies found significant changes in children’s behaviors only via observations and not from parental self-reports [30,31,38]. 

However, eight studies reported more positive parental behaviors and parent-child interactions post-intervention evaluated by direct observation by experts [29,30,31,32,34,35,37,38]. Plant & Sanders evaluated the parents and children one year after the intervention and found that at the one-year follow-up, only the children’s problematic behaviors continued to reduce but not those of the parents, and only in the SSTP-E group [37]. One study did not find any differences between the pre-and post-evaluations in any of the variables studied [33]. Another study showed a reduction in dysfunctional parenting styles through self-report questionnaires [36]. 

Of the three studies that analyzed parental sense of competence/self-efficacy [34,37,38], only two [34,37] found significant improvements between the pre-and post-evaluations. Four studies analyzed parenting stress, of which two identified reduced parenting stress levels at the post-test (also at the six-month follow-up) [35,36] while the remaining studies found no significant changes [29,37]. Relating to parental mental health, McIntyre [31] and Plant & Sanders [37] found no changes in parental depression or anxiety levels, while Williams et al. found a general increase in parental mental health levels at the post-test [38]. Finally, there were no significant changes at the post-test for conflict between parents over child-rearing [36], quality of dyadic relationship adjustment [37], and perceived social support from other parents in the group [38]. Regarding covariates and mediators, McIntyre identified the beneficial effects of having a support person present during the group intervention at the post-test (however, this effect was no longer significant when pre-test variables were considered) [30]. However, there were no significant differences in any of the studied variables between parents of children with autism and those with other DD. Williams and colleagues found that families attending more sessions achieved better outcomes and that lower parental education was associated with better outcomes [38]. Bagner & Eyberg found that pre-post changes in maternal parenting skills (increased positive parenting behaviors and decreased negative behaviors) mediated pre-post changes in child behaviors [29]. Finally, Plant & Sanders saw that adding sessions to the SSTP with content created specifically to help parents of children with DD, was more effective than the standard SSTP in producing positive changes in child behaviors while parents completed the caregiving tasks [37].

## 4. Discussion

This review aimed to advance our understanding of the key factors (e.g., formats, sample characteristics, research design) that characterized parent training interventions targeting parents of children aged 0–6 years with DD, and that could be related to treatments outcomes.

Overall, the ten interventions showed positive effects in reducing children’s challenging behaviors and negative parenting behaviors. Although each intervention presented marginally different curriculums, study designs, and main objectives, all included similar basic principles and evidence-based behavior management strategies conveyed to the parents. These interventions are based on the principles of operant and social learning theories and draw on strategies from the field of applied behavior analysis [46]. These behavioral parent training programs focus on altering the parent-child interactions so that the children’s positive behaviors are reinforced, and the reinforcement is restrained for negative or inappropriate behaviors [27]. According to the transactional models of child development, negative parent-child interactions, together with other risk factors (e.g., child development status, socio-demographic factors), may increase the likelihood of poor socio-emotional or behavioral outcomes for children [30,47,48]. Therefore, interventions aimed at supporting parents with positive parent-child interactions and behavior management strategies may help reduce the risk of developing a severe behavioral disorder or preventing one in children with DD. 

Although these are positive results in terms of the treatments’ efficacy, there remain several issues in the current body of literature that should be considered in future to corroborate these findings, and these are highlighted below.

### 4.1. Intervention Structure

In all the interventions analyzed, most parents rated the intervention sessions as helpful and were highly satisfied with the parenting skills they had learned, regardless of how the intervention was delivered (e.g., individual, group, self-administered).

None of the studies compared the same intervention delivered in different formats. Even Plant and Sanders, who tested the hypothesis that an enhanced intervention with additional modules for parents of children with DD (SSTP-E) would be superior to a standard behavioral parent training intervention (SSTP-S), found that both interventions were equally effective in producing positive changes in the child and parental behaviors [37]. Future research should examine potential differences between conducting early intervention in group-based formats and individual sessions. Moreover, future studies should examine the possibility of using a tiered approach, as in Phaneuf & McIntyre [32]. A tiered approach could help balance the program intensity with family-specific characteristics and needs. In particular, at-risk families may benefit from individual sessions while also participating in the group training sessions. 

Only one study delivered the parent training intervention through self-administered materials [32], and none explored web-based delivered methods. To date, several online parenting programs have been tested for parents with typically developed children, and although the number of experimental studies remains small, research indicates promising results in producing meaningful improvements in parenting skills and negative child behaviors [49,50,51]. Planning and verifying the efficacy of online-delivered programs in this specific clinical group would be of interest, considering their sustainability and accessibility, especially in light of the recent COVID-19 pandemic and the limitations imposed as a result of limited access to health services and professionals.

### 4.2. Methodological Limitations

Several methodological issues emerged from the studies reviewed. In terms of the study research design, only six included a control or comparison group (CG).Among them, four comprised a waitlist CG [27,35,36,37] while two interventions an “usual care” CG [30,33]. Concerning the studies with a waitlist control group, in the IG increased maternal psychological wellbeing (less stress and conflict) [35,36], increased positive interactions [27,30] and parenting positive behaviors [35,36,37] were detected compared to CG; an increase in children’s compliance and reduced children’s negative behaviors were observed as well after [27,36,37]. In one of the two studies that included a “usual care” CG, increased positive parenting behaviors and interaction were observed after the intervention [30] while in the other study, no differences between the two groups were described [33]. The limited number of studies involving a control group makes it challenging to assess the impact of interventions on the child and the parent outcomes above and beyond other intervention factors (e.g., child maturation) [27].

In addition, only four studies included follow-up assessments to examine the extent to which treatment effects were maintained over time, and the study with the longest follow-up evaluation (up to one-year post-treatment) [37] found that children’s problematic behaviors continued to reduce, but problematic parental behaviors did not. Further studies with solid research designs are needed to corroborate the efficacy of these treatments and evaluate the generalization of their effects over time. Follow-up evaluations would enable researchers and clinicians to determine the extent to which parents integrate the newly acquired strategies into the home environment [27]. 

Critical methodological issues emerged concerning the measures used to evaluate parental and children’s behaviors and the sample size. Concerning the measuring instruments, eight studies used self-report questionnaires and structural observations to evaluate the children’s behaviors [29,30,31,32,34,35,37,38] while only two studies [37,38] used self-report questionnaires and structural observations to evaluate parental behaviors. Considering the methodology used to evaluate the parental and children’s behaviors, the results from the studies included in this review are heterogeneous. Indeed, with regard to children’s behaviors, only two studies confirmed positive changes using questionnaires and observations, and two studies obtained positive results using questionnaires and observation for changes in parental behaviors. Multi-method assessments can strengthen the conclusions drawn from the results by adding ecological validity and generalization to the data; however, additional studies are needed to corroborate these findings. Also, a multi-informant approach that includes other caregivers (e.g., grandparents, teachers) could maximize the validity of the behavioral assessments and research results.

On the other hand, with few exceptions [38], the sample size of the studies we analyzed was relatively small. Small studies are essential to explore the feasibility of the methodology and piloting treatments or assessment procedures [27]. However, a small sample size can compromise the power to detect small to medium treatment effects. For this reason, future studies should implement larger randomized controlled trials to evaluate the efficacy of parent training interventions for parents of children with DD. 

### 4.3. Outcome Variables

While significant results emerged from the studies included in this review, the effects on parental mental health are less clear in terms of the efficacy of treatments to reduce children’s and parental behaviors. Of the three studies that assessed parental mental health level changes through parent reports, McIntyre found that only 20% of mothers had clinically significant reductions in depressive symptoms, while six mothers (24%) had clinically significant increases in their depressive symptoms [31]. Interestingly, Plant & Sanders found no significant changes in the pre-and post-intervention levels of anxiety and depression in participants who followed the interventions compared to the control group [37]. As the pre-intervention scores were not in the clinical range, this could explain the lack of significant post-intervention changes; some mothers may have been depressed but underreported their symptoms during the initial self-assessment phase. Conversely, weekly discussions about children’s behavioral difficulties may have focused more attention and awareness on the home situation, exacerbating the depressive symptoms in some mothers. Williams and colleagues also found an overall increase in the mental health problems of participants who followed the intervention [38]. However, the lack of a comparison to a control group makes it impossible to conclude that these changes were a direct result of participation in the intervention. 

Previous research provided substantial evidence that parents of children with DD experience higher levels of stress and depression than those of typically developing children [52]. Future studies are needed to investigate the efficacy of parent training interventions on parental mental health, including parents in the clinical range for stress or depressive symptoms, and testing the efficacy of additional treatment components to directly address coping, stress, and depression in families with children with DD [31]. Additionally, since these interventions primarily involve mothers, future studies should test the generalization of the results to their partners, considering that maternal and paternal mental health impact child behavior [53,54]. 

Given the importance of parental mental health on family wellbeing, future research should evaluate parental mental health as an outcome variable and as a factor intervening in the effectiveness of the intervention. Previous studies have shown that low levels of parental mental health adversely affect parent–child attachment and parental perceptions of the difficulties and demands of caregiving [53]. Moreover, factors not associated with the parenting intervention could have impacted changes in parental depression and mental health scores. For example, marital discord [55,56] and financial problems [57] have been shown to be related to maternal and paternal depression. In our review, only Bagner & Eyberg found that pre-post changes in maternal parenting skills (increased parenting positive behaviors and decreased negative ones) mediated pre-post changes in child behaviors [29]. It could be hypothesized that parental training interventions effectively reduce children’s challenging behaviors because of changes in parenting and parent-child interactions. As these assumptions have been rarely tested in the DD parent training literature [27]), further studies are needed to analyze the mediation and/or moderation role of parenting and parental mental health in the relationship between the interventions and outcomes. 

Socio-demographic variables should also be considered when planning and implementing parenting interventions. One of the included studies found that lower parental education is associated with better outcomes [38]. Even if mothers with lower levels of education are less likely to access healthcare services for their children [58], children from lower SES backgrounds and their mothers gain more from participating in early intervention programs than those from higher SES backgrounds [38,59]. 

Finally, among the studies considered, one measured social support as an outcome variable [38]. Authors describe that after the *Sing & Grow* intervention, the parental perception of social connection increased between parents and professionals and among the participating parents (*ibidem*). Cooley describes the presence of both formal and informal types of social support. Formal support is usually offered by professionals or organizations, whereas informal support is commonly offered by other family members, peers, and individuals who may share similar life experiences [60]. Concerning parenting children affected by DD, the literature widely recognized the essential role of social support at all stages of life [61,62,63]. Parental perception of a successful support network is associated with better adaptation to the diagnosis process and the implementation of more effective coping strategies. Furthermore, increased psychological wellbeing, lower levels of anxiety and depression, and improved quality of life are associated with the perception of effective support, especially for mothers [62]. In a recently published contribution, Cutrona and Russell describe a new conceptualization of the construct of social support, outlining its role in sustaining the individuals’ personal growth [64]. This assumption appears especially true for parents of children with DD. Parenting can be challenging and is characterized by considerable effort and strain to sustain their children’s developmental pathways, especially during the early stages of life. In some cases, intensive support may be required up to adulthood, making these relationships even more significant [20]. Considering the risk factors involved in parenthood, interventions that address social support would further benefit these families [65]. In addition, professional support measures could actively involve other significant secondary caregivers who contribute to children’s everyday care other than the parents, such as grandparents or other meaningful family members. Future perspectives may comprise specific evidence-based training for secondary caregivers who represent an important resource for parents and children. 

The findings of this review should be interpreted in light of the limitations of our work. First, we only assessed the English-language literature and may, therefore, have overlooked significant findings reported in other languages. Second, although we strove to conduct an exhaustive search, it is possible that a relevant search term may have been omitted, and consequently that some relevant studies were not retrieved. In addition to this, the narrative nature of our review precluded us to make a quantitative comparison of the studies’ characteristics and results. Future studies should implement this work also including a meta-analysis. Nonetheless, to the best of our knowledge, this review is the first to systematically review parent training programs targeting parents of children with different DD aged 0–6 years.

## 5. Conclusions

In conclusion, this review underlines a range of critical issues that should be considered in future studies to enhance the efficacy of behavioral parent training programs with parents of children with DD. The 10 studies in this systematic review generally show positive outcomes for parents and children’s behaviors. However, conclusions from these studies are limited by the small sample sizes, lack of control or comparison groups, the absence of follow-up evaluations, the absence of a multi-method assessment, and the lack of multiple outcome measures. Furthermore, larger-scale studies that are adequately powered should explore the role of covariates, mediators, and moderators that account for changes in the dependent measures. It is essential to examine the covariates, moderators, and mediators, as these are vital to enhancing our knowledge of intervention effectiveness for sub-populations and understanding the underlying mechanisms of treatment outcomes [27].

Finally, given that the 10 studies reviewed were all conducted in the USA, Australia, and China. Concerning USA, several Federal legislative efforts addressed children and youths with disabilities. The Administration on Disabilities (AoD) is an operating division of the U.S. Department of Health and Human Services that actively collaborates with each state and local community to build network and foster crosswise and adequate support. Under the provisions established through various authorizing statutes, AoD aims to improve “opportunities for people with disabilities to access quality community services, experience equality, equity, and inclusion in all facets of community life” (https://www.cdc.gov/ncbddd/developmentaldisabilities/index.html, accessed on 1 May 2022). 

Australia, historically evolved into a unique social protection and welfare system supporting community and families; now it bears the hallmarks of policy development in the United States, with which it most identifies [66]. Concerning China’s active welfare policies, enormous changes in public policy for people with disabilities were recently observed. In the past decades, some of the most progressive disability-related legislation in the world was established. In spite of this promising evidence, as dramatically pointed out by Kwok et al. [67], the actual experiences of people with disabilities have not improved and a substantial disconnection between the legislation and implementation of policy is observed (*ibidem*).

It would be of interest to investigate parent training interventions for parents of children with DD in other countries (e.g., Europe) in the future. As suggested by Kong and Au, differences in parenting beliefs and practices may influence the adoption of parent training programs across culturally diverse populations [35,68].

## Data Availability

Not applicable.

## Appendix A

**Table A1:** Participants and Intervention Characteristics.

Authors, Year (Country)	Intervention	Parents (No.)	Children (Age; M (SD))	SES	Diagnosis	RCT	FU	Duration	Individual/ Group-Based	Main Contents	Activities & Materials	Homework	CG
Bagner & Eyberg, 2007 (USA) [27]	PCIT (Eyberg & Child Study Lab, 1999) [69]	30 Mo.; IG = 10; CG = 12; 5 Mo study dropouts, 3 Mo waitlist dropout	IG = 52.40 (8.81) m.; CG = 55.87 (11.38) m.	-	Mild or moderate MR + ODD	yes	no	12 w. CDI = 5 sessions; PDI = 7 sessions 1 hr. per w.	Individual	CDI = enhancing the parent–ch. relationship, increasing positive parenting, improving ch. social skills; PDI = improving parents’ ability to set limits and follow through consistently to reduce ch. Noncompliance and disruptive behav.; In the last few sessions, parents learn variations of the PDI procedure to deal with aggressive behav. and public misbehav.	Therapists actively coach parents toward mastery of the interaction skills	no	Waitlist
Barton & Lissman, 2015 (USA) [34]	IYPT (Webster-Stratton, 2001)-adapted version [24]	2 Mo.	48 m. & 53 m.	-	Dev. delays (communication, cognitive and social skills) + eligibility for special education services + Ch. challenging behav. + at least one parenting risk factor	no	6–10 w.	8 w., 2 hr. per w. + 2 Ind. coaching sessions for 12 w., and 1 for 4 w., 45 min.-1 h each	Group + Individual	Group: Acknowledge appropriate behav. with specific praise, help child. understand expectations and respond appropriately to challenging behav. Individual: 15 min. obs. of parent and child. behav. + feedbacks;	Video vignettes, revision of homeworks, problem solving discussions, handouts	yes	no
Boyce et al., 1993 (USA) [33]	PIE curriculum	51 families; IG = 26 (92% intact families) CG = 25 (96% intact families)	IG = 47.2 (9.7) m.; CG = 47.1 (8.1) m.	Upper-middle class	Moderate-severe DD (Diagnoses: general dev. delay, Down syndrome, motor, sensory, and behav. impaired)	yes	no	IG = 15 w., 90 min. per w. CG = 15 w., 5 days per w., 2.5 hr. each	Group (8–10 parents per group)	Teach parents strategies they could implement at home, prepare parents to interact more effectively with professional intervenors, teach basic ch. dev. principles, provide an opportunity for parents to discuss challenges associated with parenting a ch. with a disability	Lectures, discussions, and demonstrations	yes	Ch. Received instructions from a certified special education teacher and therapist in dev. areas (motor, language, self-help, cognitive, social skills)
Kong & Au, 2018 (China) [35]	IYPT (McIntyre, 2008b [31]; Webster-Stratton, 2007) [70]-cultural adaptation	47 parents; IG = 25 (19 Mo.); CG = 22 (21 Mo.)	IG = 55.92 (10.88) m.; CG = 56.52 (11) m.	Heterog.; half families receiving government subsidies	DD (ASD, Dev. delay, ADHD, PDD, ID)	yes	no	12 w., 2 hr. per w.	Group	Teaches parents to acknowledge appropriate behav. with specific praise, help ch. understand expectations, and respond appropriately to challenging behav.	Video vignettes, revision of homeworks, problem solving discussions, handouts	yes	Waitlist
Leung et al., 2013 (China) [36]	Triple P Level 4 (Standard) (Sanders, 1999) [42]	81 parents; IG = 42 (37 Mo.); CG = 39 (35 Mo.)	IG = 50.48 (12.31) m.; CG = 49.74 (10.71) m.	-	Physical dis., ASD, dev. delay	yes	6 mo.	8 w., 6 sessions of 2 hr. each + 2 telephone sessions	Group + Individual	Group: Teaches parenting strategies to enhance the quality of parent-ch. relationships, encourage desirable behavior, teach new skills, and manage misbehav., enhance parental self-regulation Individual: 2 telephone sessions at follow-up	Mini lectures, discussions, role play and exercises, workbook	yes	Waitlist
McIntyre, 2008a (USA) [30]	IYTP for parents of children with DD (McIntyre, 2008b) [31]	44 parents IG = 21 (19 Mo.); CG = 23 (20 Mo.)	IG = 4.11 (1) y.; CG = 3.68 (0.8) y.	Heterog.;	Mild-moderate DD (45–85 score on the VABS)	yes	no	12 w., 2.5 hr. per w.	Group (8–12 parents per group)	Teaches parents to acknowledge appropriate behav. with specific praise, help child. understand expectations and respond appropriately to challenging behav.	Video vignettes, revision of homeworks, problem solving discussions, handouts	yes	Usual care
McIntyre, 2008b (USA) [31]	IYTP for parents of children with DD	25 parents (23 Mo.)	3.99 (0.87) y.	Heterog.;	Mild-moderate DD (45–85 score on the VABS);	no	no	12 w., 2.5 hr. per w.	Group (8–12 parents per group)	Teaches parents to acknowledge appropriate behav. with specific praise, help child. understand expectations, and respond appropriately to challenging behaviors	Video vignettes, revision of homeworks, problem solving discussions, handouts	yes	no
Phaneuf & McIntyre, 2011 (USA) [32]	IYPT for parents of children with DD (McIntyre, 2008b) [31]-adapted	6 Mo.	4.11 (0.77) y.	-	Mild-moderate DD (45–85 score on the VABS); (Diagnoses: ASD, speech/ language delays, and global dev. delays) + Observed parent–ch.int. characterized by > 20% of intervals containing negative parenting strategies	no	3 m.	Tier 1 = 3 w.; Tiers 2 = 11 w., 2.5 hr. per w.; Tier 3 = 1 session	Tier 1 = self-administered; Tier 2 = Group; Tier 3 = Individual;	Tier 1 and Tier 2: teaches parents to acknowledge appropriate behav. with specific praise, help child. understand expectations, and respond appropriately to challenging behaviors Tier 3: video-feedback session at home	Tier 1: audiotapes/CDs and reading materials from the IYPT; Tier 2: Video vignettes, revision of homeworks, problem solving discussions, handouts	yes	no
Plant & Sanders, 2007 (Australia) [37]	SSTP-S (Roberts et al., 2006) & SSTP-E [71]	74 families SSTP-S = 26 SSTP-E = 24 CG = 24	SSTP-S = 54.26 (15.25) m.; SSTP-E = 56.63 (12.36) m.; CG = 54.04 (13.16) m.	Heterog.;	DD borderline-severe (VABS) (Diagnoses: ASD, Global Dev. Delay, Down Syndrome, chromosomalabnormality other than Down Syndrome, Cerebral Palsy) + Ch. challenging behav. (ECBI; intensity score 131 or problem score 15)	yes	1 y.	SSTP-S = 10 weeks; SSTP-E = 16 weeks; 60–90 min. per w.	SSTP-S & SSTP-E: Group + Individual	Group SSTP-S: strategies to promote child competence and dev.; enhance generalization and maintenance of parenting skills; SSTP-E: SSTP-S + training related to grief and loss issues, stress and coping, time management, working collaboratively with professionals, and strengthening social supports Individual: 2 sessions were conducted in the family home where parents were observed implementing parenting skills with their ch. and received feedbacks on their strengths and weaknesses	Modelling, role plays, feedbacks, use of specific homework tasks, workbook; SSTP-E: additional active skill training and support for parents to cope with caring for a ch. with a DD	yes	Waitlist
Williams et al., 2012 (Australia) [38]	Sing & Grow (Nicholson et al., 2008) [44]	201 dyads mother-child.	3–60 mo.; 34.4 mo. (mode = 48 mo.)	-	DD (Diagnoses: general, usually global, dev. delay; Down syndrome; other syndromes or specific diagnoses including brain injury, fetal alcohol syndrome, and rare chromosomal abnormalities, ASD, speech and language impairment, cerebral palsy, sensory impairment)	no	no	10 w., 1 hr. per w.	Group (8–10 parents per group)	Music therapy intervention which encourages social responsiveness, practice of fine and motor skills and concept comprehension, following simple instructions, turn-taking and sharing, encourage physical touch, closeness and bonding, use of praise, modelling and positive reinforcement	Musical activities and CDs and song books to transfer the activities to the home environment	no	no

Note. ADHD = Attention Deficit Hyperactivity Disorder; ASD = Autism Spectrum Disorder; Ch. = child/children; CG = Control group; Dev. = Developmental; dis. = disability; DD = Developmental disabilities; ECBI = Eyberg Child Behavior Inventory FU = Follow-up evaluation; Heterog. = heterogeneous; hr. = hour; ID = Intellectual disability; IG = Intervention group; int. = interaction(s); IYPT = The Incredible Year Parent Training; m. = months; M = means; min.= minutes; Mo. = mothers; MR = mental retardation; No. Number of participants; ODD = Oppositional defiant disorder; PCIT = Parent-Child Interaction Therapy; PIE = Parents Involved in Education; PDD = Pervasive Developmental Disorder; RCT = randomized controlled trial; SD = standard deviation; SSTP-E = Stepping Stones Triple P-Positive Parenting Program-Enhanced; SSTP-S = Stepping Stones Triple P-Positive Parenting Program-Standard; VABS = Vineland Adaptive Behavior Scale; w. = week(s); y. = year(s).

**Table A2:** Interventions’ outcome variables, measures and results.

Authors, Year (Country)	Intervention	Outcome Variables (Measure)	Observation Methodology	Covariates	Mediators/Moderators	Results
Bagner & Eyberg, 2007 (USA) [27]	PCIT (Eyberg & Child Study Lab, 1999) [69]	Ch. Behav. (Q.); Frequency of disruptive Ch. behav. (Q.); Parenting stress (Q.); Parent behav. and ch. compliance (Obs.)	Validated observational recording system; Led by experts	no	Pre-post treatment changes in maternal parenting skills	TG compared to CG: >maternal positive int.; <negative ch. behav. reported by Mo.; >ch. compliance; >positive parenting behav. and <negative behav. during Mo.-ch.int. mediated changes in ch. behav.
Barton & Lissman, 2015 (USA) [34]	IYPT(Webster-Stratton, 2001)-adapted version [24]	Positive parenting practices (Obs.); Ch. challenging behav. (Obs.); Ch. socioemotional dev. and parental concerns about ch. dev. and challenging behav. (Q.); Parental sense of competence/self-efficacy (Q.)	Ad hoc partial interval observational recording system; Led by experts	no	no	>positive parent behav.; 11/21 and 13/21 coaching sessions without ch. challenging behav.; <ch. challenging behav. reported by mothers; >sense of parental competence
Boyce et al., 1993 (USA) [33]	PIE curriculum	Ch. dev. (Dev. test by experts; Q. by parents and teachers); Intellectual abilities (Dev. test by experts); Family functioning (Q.); Parental positive behav. (Obs.); Educational program and parental participation (Q. by teachers)	Validated observational rating system; Led by experts	Mo. Y. of education; Fa. hours of work; Ch. general health; Changing in family living situations; Family resources; Stressful life events;	no	No differences between the TG and CG
Kong & Au, 2018 (China) [35]	IYPT (McIntyre, 2008b [31]; Webster-Stratton, 2007) [70]-cultural adaptation	Quality of parent-ch.int. (Obs.); Ch. joint attention (Obs. adapted); Parental verbal responsiveness (Obs. adapted); Parenting stress (Q.); Ch. behav. (Q.);	Videotaped; Validated observational rating system; Led by experts	no	no	TG compared to CG: <parenting stress; <ch. behav. problems reported by parents >labelled praise; >dialogues more positive, ch.-centred and sensitive to child needs; >ch. joint attention
Leung et al., 2013 (China) [36]	Triple P Level 4 (Standard) (Sanders, 1999) [42]	Ch. disruptive behav. (Q.); Parental stress (Q.); Dysfunctional discipline styles in parents (Q.); Conflict between partners over ch.-rearing (Q.);	-	no	no	TG compared to CG: <ch. behav. problems (also at 6 mo. follow-up); <parental stress and conflict (also at 6 mo. follow-up); <dysfunctional parenting styles (laxness and over-reactivity; alsoat 6 mo. follow-up);
McIntyre, 2008a (USA) [30]	IYTP for parents of children with DD (McIntyre, 2008b) [31]	Ch. Behav. problems (Q.); Family impact of the ch. (Q.) Parent-ch. interactions quality (Obs.)	Videotaped; Ad hoc observational rating system; Led by experts	Presence of a support person during the intervention; type of DD (autism vs. other DD)	no	TG compared to CG: <negative and inappropriate parent-ch. int.; <negative ch. behav. problems; beneficial effects of having a support person present at post-treatment but no more sign. when considering pre-treatment assessment; No diff. between autism ch. and ch. with other DD
McIntyre, 2008b (USA) [31]	IYTP for parents of children with DD	Ch. behav. problems (Q.); Family impact of the ch. (Q.); Parent-ch. int. quality (Obs.); Parental depression (Q.)	Videotaped; Ad hoc observational rating system; Led by experts	no	no	<inappropriate parental behav.; >perceived positive impact of the ch.; no pre-post differencesin parental perceived negative ch. behav. And in the negative impact of the ch. on the family; <observed maladaptive ch. behav.;
Phaneuf & McIntyre, 2011 (USA) [32]	IYPT for parents of children with DD (McIntyre, 2008b) [31]-adapted	Parent-ch. int. (negative and positive parenting strategies + ch. problem behav.) (Obs.) Ch. problem behav. (Q.)	Videotaped; Ad hoc observational rating system; Led by experts	no	no	= or <observed percentages of negative parenting strategies at 3 mo. FU in comparison to post-treatment; all six Mo. engaged in positive strategiesduring more than 40% of the intervals observed; < negative ch. behav. both from Obs. and Q.
Plant & Sanders, 2007 (Australia) [37]	SSTP-S (Roberts et al., 2006) & SSTP-E [71]	Parent-ch. int. (parent and ch. behav.) (Obs.); Ch. behav. problems (Q.); Frequency of difficult ch. behav. when completing caregiving tasks (ad hoc Q.); Frequency of problematic caregiving tasks (ad hoc Q.); Dysfunctional discipline styles in parents (Q.); Parental sense of competence (Q.); Parental depression, anxiety, and stress (Q.); Quality of dyadic relationship adjustment (Q.)	Videotaped; Validated observational rating system; Led by experts	no	no	TG compared to CG: >positive changes in ch. behav.; >adaptive parenting skills; >parental competence; No changes in parental stress and adjustment; SSTP-E more effective in producing positive changes in ch. behav. during parents’ completion of caregiving tasks than SSTP-S; At 1 y. FU only ch. problematic behav. continue to reduce but only in SSTP-E
Williams et al., 2012 (Australia) [38]	Sing & Grow (Nicholson et al., 2008) [44]	Parental mental health (Q.); Parental self-efficacy (adapted Q.); Parent-ch. int. (responsiveness, irritable parenting, parent engagement in learning activities at home) (Q.); Ch. behav. (adapted Q.); Quality of parent and child. behav. (Obs.) Social support with the other parent in the group (ad hoc Q.);	Validated observational rating system; Led by experts	Ch. age and gender; Main language used at home; Parental low education level; Main income; Single parent; High attendance (6 or >sessions)	no	>parent-ch. positive int.; >parental mental health, ch. communication and social skills; >ch. behav. observed but not perceived by parents; >parenting sensitivity observed but not reported by parents; families who attended more sessions achieved better outcomes; lower parental education is associated with better outcomes

Note. Behav. = behavior(s); Ch. = child/children; CG = Control group; Dev. = Developmental; DD = Developmental disabilities; Fa. = Father(s); FU = Follow-up evaluation; int. = interaction(s); IYPT = The Incredible Year Parent Training; m. = months; Mo. = mothers; Obs. = Observation; PCIT = Parent-Child Interaction Therapy; PIE = Parents Involved in Education; SSTP-E = Stepping Stones Triple P-Positive Parenting Program-Enhanced; SSTP-S = Stepping Stones Triple P-Positive Parenting Program-Standard; Q. = Questionnaire(s); y. = year(s).
